# The avian taste system

**DOI:** 10.3389/fphys.2023.1235377

**Published:** 2023-09-08

**Authors:** Shahram Niknafs, Marta Navarro, Eve R. Schneider, Eugeni Roura

**Affiliations:** ^1^ Centre for Nutrition and Food Sciences, Queensland Alliance for Agriculture and Food Innovation, The University of Queensland, St Lucia, QLD, Australia; ^2^ Department of Biology, University of Kentucky, Lexington, KY, United States

**Keywords:** avian species, gustatory system, taste receptors, diet, preference

## Abstract

Taste or gustation is the sense evolving from the chemo-sensory system present in the oral cavity of avian species, which evolved to evaluate the nutritional value of foods by detecting relevant compounds including amino acids and peptides, carbohydrates, lipids, calcium, salts, and toxic or anti-nutritional compounds. In birds compared to mammals, due to the relatively low retention time of food in the oral cavity, the lack of taste papillae in the tongue, and an extremely limited secretion of saliva, the relevance of the avian taste system has been historically undermined. However, in recent years, novel data has emerged, facilitated partially by the advent of the genomic era, evidencing that the taste system is as crucial to avian species as is to mammals. Despite many similarities, there are also fundamental differences between avian and mammalian taste systems in terms of anatomy, distribution of taste buds, and the nature and molecular structure of taste receptors. Generally, birds have smaller oral cavities and a lower number of taste buds compared to mammals, and their distribution in the oral cavity appears to follow the swallowing pattern of foods. In addition, differences between bird species in the size, structure and distribution of taste buds seem to be associated with diet type and other ecological adaptations. Birds also seem to have a smaller repertoire of bitter taste receptors (T2Rs) and lack some taste receptors such as the T1R2 involved in sweet taste perception. This has opened new areas of research focusing on taste perception mechanisms independent of GPCR taste receptors and the discovery of evolutionary shifts in the molecular function of taste receptors adapting to ecological niches in birds. For example, recent discoveries have shown that the amino acid taste receptor dimer T1R1-T1R3 have mutated to sense simple sugars in almost half of the living bird species, or SGLT1 has been proposed as a part of a T1R2-independent sweet taste sensing in chicken. The aim of this review is to present the scientific data known to date related to the avian taste system across species and its impact on dietary choices including domestic and wild species.

## 1 Introduction

Birds are the most diverse group of vertebrates with more than 10,000 species (Pimm et al., 2006). Birds compared to mammals have often been referred to as having a poor taste acuity based on low numbers of taste buds (e.g., 767 in chicken compared to 7,902 in humans), less saliva production, and rapid transit of food through the oral cavity (Klasing, 1998; Roura et al., 2013). This undermining of the avian taste not only neglects the millions of years of evolution of the chemical senses, but it also underestimates the existence of a highly developed taste (gustatory) system crucial for the adaptation of birds to a diverse range of ecosystems and dietary regimes. Evolutionary analyses based on taste receptor genes showed that the pillars of gustation were developed in vertebrates before the separation of teleost fish and tetrapods which ultimately led to birds and long after to mammalians. In addition, sweet and umami related taste receptors have remained highly conserved in vertebrates including humans (Shi and Zhang, 2006). Intriguingly, it appears that the entire avian clade evolved based on the early loss of sweet taste receptor (T1R2). However, soon after the loss, sugar detection ability was evolved by shifting umami taste receptors (T1R1-T1R3) to sweet sensor in songbirds comprising almost half of all current bird species (Toda et al., 2021). In fact, in two-choice assays, nectarivorous and non-nectarivorous songbirds strongly preferred sucrose solution over water. Thus, regardless of their diet, songbirds have the ability to perceive sweet taste (Toda et al., 2021).

Taste perception plays a key role in sustaining the adequate consumption of a balanced diet. In the wild, dietary choices reflect the innate drive to achieve and maintain nutritional homeostasis (Roura and Navarro, 2018). This, in turn, implies the existence of a network of nutrient receptor/sensors covering physiological functions starting with the control of appetite, dietary selection and overall feed intake (Roura et al., 2019). Thus, the main role of the taste system in the oral cavity is to perceive dietary nutrients and assess the nutritional quality of the meal. The avian sense of taste has been tuned to distinguish at least five and up to seven groups of tastants (or nutrients) including amino acids, fatty acids, salts, acids, bitterants and potentially simple carbohydrates and calcium (Roura et al., 2013; Niknafs and Roura, 2018).

The avian taste system has been studied by researchers from two perspectives. Firstly, understanding the biological and ecological aspect of taste mainly done by ornithologists. These scientists have investigated a wide range of wild and domesticated birds. Bath (1906) was among the pioneers of this area investigating the taste system in blackbird, barn swallow, mallard, flamingo, budgerigar, oystercatcher, European greenfinch, and common (European) starling. Secondly, the poultry scientist’s perspective trying to understand agricultural and economic implications of the avian taste system. Poultry species such as chicken, turkey, quail, and duck have been the main interest. These scientists try to understand how the taste system plays a role in feed intake regulation, opening opportunities for using non-conventional feedstuff to the poultry industry (Roura et al., 2013).

This review article will focus on the description of the avian taste system including a brief historical perspective, fetal development, anatomical structure and function, oral-brain axis, molecular mechanisms eliciting taste and behavioral aspects linked to oral nutrient sensing in avian species. Some of the diversities observed between bird species relevant to the taste system will be highlighted. It is noted that the description of taste types (sweet, umami, bitter, salty, and sour) is necessarily based in anthropomorphic descriptions. Admittedly, the taste qualities elicited by taste receptor ligands is unknown in non-human animals. This review is based on the assumption that homology in taste receptor genes relates to homology in the type of the taste perceived.

## 2 A brief historical perspective on the discovery of the avian taste system

The study of the avian taste system has been ongoing at least since the 19th century. However, the first traceable scientific study failed to identify taste papillae or other anatomical structures such as taste buds known to exist in mammalian tongues (Merkel, 1880). In 1903, Elliott Coues in the fifth edition of his book, *Key to North American Birds*, laid out the importance of avian taste in food choice and the involvement of chorda tympani and cranial nerves in sensing taste (Coues, 1903). However, there was no mentioning of taste buds in birds. The discovery of avian taste buds should probably be credited to Eugen Botezat in 1904 (Botezat, 1906). Soon after, a topographical study on taste buds across several bird species was published by Bath (1906). After these first discoveries, no searchable scientific research on the avian taste system was published until the end of World War II. Some of the early reports after World War II on chickens showed the existence of a small number of only eight taste buds in the oral cavity (Lindenmaier and Kare, 1959). Consequently, a consensus that taste in birds did not have the functional relevance that it had in mammals dominated the scientific community until recent times (Niknafs and Roura, 2018).

A more accurate understanding of the relevance of the avian taste system started with the work published by Berkhoudt (1985) and especially by Ganchrow and Ganchrow (1985), who reported 70 and 316 taste buds in the chicken oral cavity, respectively. Ganchrow and Ganchrow reported that 69% of the buds were on the palate and not on the tongue like in mammals. These findings not only confirmed some of the earlier observations in wild birds reported by Bath (1906) but were also a turning point that triggered novel interest in the sense of taste in chickens. Some of the research published in the following years illustrated that birds had the ability of making dietary choices based on taste to a similar or higher accuracy than mammals (Matson et al., 2000). From the behavior point of view, a series of studies published by Van Heezik et al. (1983) and Gerritsen et al. (1983) demonstrated strong evidence that taste cues are used by shorebirds including Sanderling (*Calidris alba*), Dunlin (*C. alpina*), Purple Sandpiper (*C. maritima*), and Red Knot (*C. canutus*) to locate their prey and regulate their foraging behavior.

The advent of the genomic era triggered a positive boost on the appreciation of the taste system in avian species. In 2004, the first bird species genome, the chicken, was sequenced and released (Hillier et al., 2004). Analyzing the chicken genome revealed a full repertoire of taste receptor (TR) genes but also the lack of the mammalian sweet taste receptor T1R2 and a smaller number of bitter taste receptors (T2R), consisting of only three members compared to 25 in humans (Shi and Zhang, 2006). In addition, taste buds were historically studied using microscopic methods and the focus of the studies was mainly on tongue (Ganchrow and Ganchrow, 1985). However, recently molecular and immunohistochemistry (IHC) approaches along with expanding the search to palate led recently to the discovery of a plethora of taste buds in the oral cavity of chickens. IHC enables researcher to detect sensory cells using florescent antibodies resulting in identifying taste buds that have been missed using microscopic visualization. Rajapaksha et al. (2016) reported 767 taste buds in chicken, 66% of which on the palate and the rest on the base of the oral cavity. In the past few years, fascinating aspects of the avian taste system have been discovered which highlight the evolutionary relevance of the avian taste system to adapt to dietary requirements in several bird species (Baldwin et al., 2014; Toda et al., 2021; Cockburn et al., 2022). Looking to the future, the recent release of the whole genome sequence of 363 bird species in 2020 and the initiative to sequence the genome of all the 10,000 bird species (B10K project: https://b10k.genomics.cn) will provide the opportunity to further understand the role of taste system in avian biology and evolution (Feng et al., 2020).

## 3 Comparative anatomy and development of the avian taste system

One of the most characteristic body structures that differs amongst bird species is the beak/bill. Beaks have been classified to reflect the adaptation to feeding regimes. Similarly, the avian tongue is also highly variable in length and shape adapted to food collection, manipulation, and swallowing (Berkhoudt, 1992). Beaks and tongues and their anatomical structure and function are highly associated with the topographic distribution of taste buds in the oral cavity in avian species (Berkhoudt, 1992; Kudo et al., 2008). For example, Crole and Soley (2015) demonstrated that taste buds are strategically located in the non-pigmented oropharynx in *Dromaius novaehollandiae*, enabling the bird to sample the food during ingestion.

### 3.1 Embryonic taste development

In chickens, taste bud development begins at early stages of embryonic development, and the rapid formation of taste buds occurs during the last stages between days 17 and 21 (Ganchrow and Ganchrow, 1985). In chicken embryos, beak and tongue can be differentiated by day 8, at the same time, mandibular salivary glands start developing from mucosal stem cells concluding on day 16 (Hamilton, 1953; Hamburger and Hamilton, 1992). In contrast, taste buds start emerging later at day 17 in the base of the epithelium forming spherical cluster of cells in the lower beak (Ganchrow and Ganchrow, 1987). These first buds reach the surface of the epithelium by day 19 when taste pores become distinguishable (Ganchrow and Ganchrow, 1987). On embryonic day 20, basal and perigemmal cells in the taste buds are recognizable (Ganchrow and Ganchrow, 1989). By the time of hatch at day 21, taste buds continue to elongate to an ovoid shape and almost all buds’ pores are opened to the oral cavity with no spherical shapes remaining (Ganchrow and Ganchrow, 1987). Before hatching, the embryonic taste system is responsive to stimuli such as quinine, fructose, HCl, NaCl, and KCI (Vince, 1977). At hatch, taste buds are fully functional and responsive to taste stimuli. There is a fast increase in the number of taste buds during embryonic day 17 and 18 and reaches 80 taste buds by day 19 (Cheled Shoval et al., 2022). While some data suggest that the total number of taste buds has been reached before hatching, some other have shown that the taste system continues to grow and mature reaching the peak by day 3 post-hatching (Ganchrow and Ganchrow, 1987; Ganchrow et al., 1995; Rajapaksha et al., 2016). These inconsistencies between research groups may indicate potential differences in the development of taste buds between breeds and sexes (Liu et al., 2018).

### 3.2 Oral topographic distribution of taste buds

Unlike mammals, the avian tongue is not a major sensory organ. The lingual epithelium is often keratinized and does not contain differentiated appendices or organelles such as taste papillae (Elner et al., 2005). Most taste buds in birds are located on the soft and glandular epithelia of the palate (Erdoğan and Iwasaki, 2014). In addition, taste buds appear in clusters around salivary ducts as it has been shown in many avian species including chicken, sparrow, kingfisher, spotted owl, pigeon hawk, western sandpiper, dunlin, and parrot (Nalavade and Varute, 1977; Elner et al., 2005). The presence of abundant saliva in the oral cavity is crucial to facilitate the sensing of food compounds by reaching the taste buds and getting in physical contact with the taste receptors. The production of saliva is variable depending on feeding strategies being best developed in birds that consume dry diets such as granivores or insectivores (Beltman and Kare, 1961). However, avian species have often been described as having limited saliva secretion when eating (Klasing, 1998). Thus, pattern of location of taste buds surrounding salivary glands guarantees an efficient use of the saliva for taste sensing (Kurosawa et al., 1983).

**FIGURE 1:**
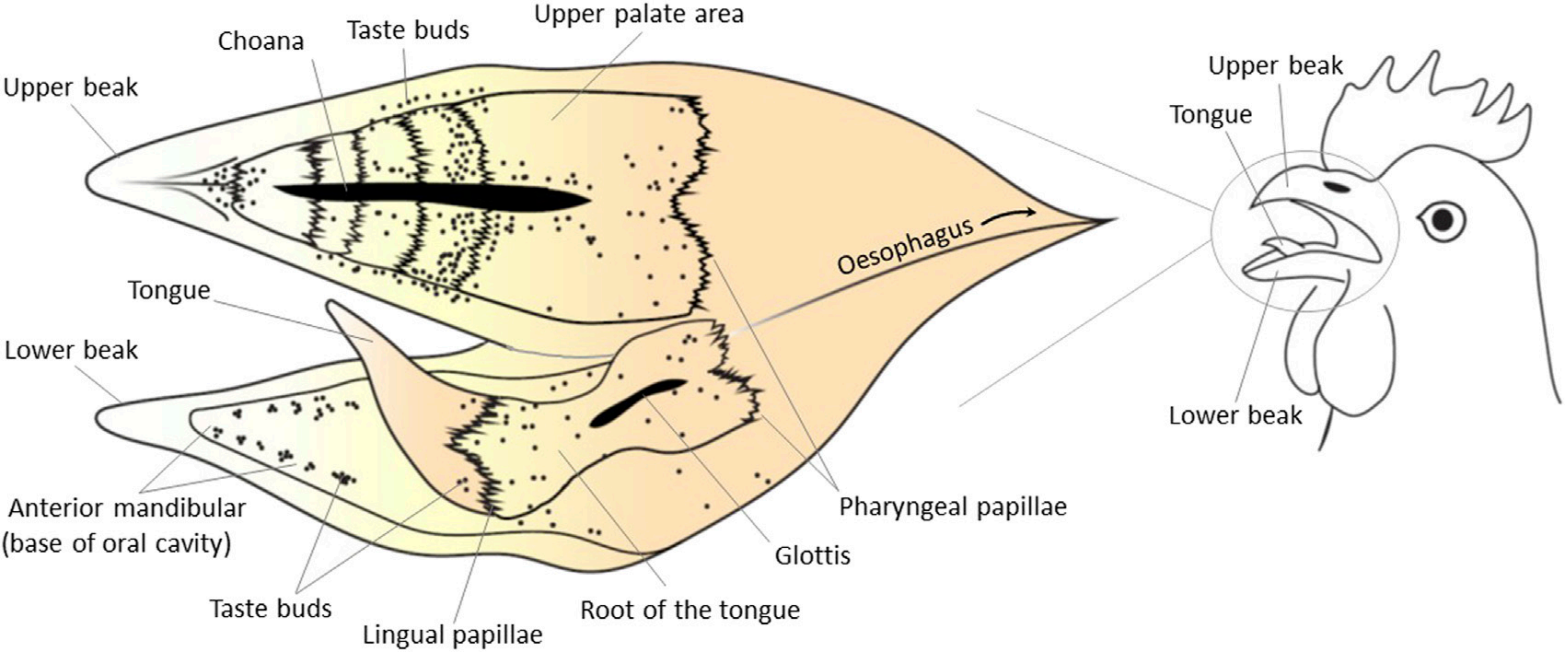
Topographical distribution of taste buds in the chicken oral cavity. The schematic view of the internal surface of the oral cavity is shown as a flat surface with the upper and lower surfaces next to each other in an “open book”-type view. Black dots represent taste buds. Lingual and/or pharyngeal papillae refer to mechanical (and not taste) organelles. The tip of the tongue is keratinized and does not contain taste buds. The main density of taste buds is in the upper palate. Other parts of the oral cavity in chickens with presence of taste buds include the root of the tongue and oropharynx, the base of the tongue and the soft oral mucosa in the mandible on both sides of the tongue. Taste buds gather in groups of 1–10 to form clusters, and these clusters are broadly distributed on the palate and the base of the oral cavity mainly around salivary ducts. This figure was created based on the data published by Kudo et al. (2008), Rajapaksha et al. (2016) using Adobe Illustrator 24.0.

The topographic distribution of taste buds in the avian oral cavity has been related to feeding behaviors and ingestion routes reflecting the main role of the tongue in food collection and swallowing (Martin, 2017). Three distribution patterns can be differentiated related to tongue functions (King and McLelland, 1984; Klasing, 1998):

1 Type I relates to tongues adapted to swallowing. These tongues are characteristically short and non-protrusile. This type has been described in chicken, pigeon, pelicans, cormorants, ostriches, or cassowaries. For example, the chicken tongue is keratinized in the tip and central body but not in the back towards the pharynx (Berkhoudt, 1992). Tongue keratinization seems incompatible with sensory properties. The process of swallowing involves pecking the food between beak tips before moving it intraoropharyngeal with the tongue pressing the food against the taste buds on the upper palate. These movements optimize the contact of food particles with the saliva and taste sensory cells (Fowler, 1991; Van den Heuvel and Berkhoudt, 1997). The taste bud distribution in chicken (Figure 1) seems to be consistent with the swallowing process. In brief, the major location of taste buds in this group is the upper palate (almost 70%), but they are also found in the strips of soft oral mucosa on both sides (5%), at the back (5%), and base (20%) of the tongue (Bath, 1906; reviewed by Berkhoudt, 1992; Rajapaksha et al., 2016). However, there is a wide range of diversity even within this category. For example, the Eurasian collared dove (*Streptopelia decaocto*) and chicken have similar tongue anatomy, but taste buds and salivary glands are present in the lingual epithelia in Eurasian collared dove. This has been claimed to be an adaptation to an herbivorous feeding style. The body and root of the tongue and the laryngeal mound contain ovoid-shaped taste buds (El-Mansi et al., 2021).
2 Type II refers to tongues adapted for food manipulation. These tongues are usually not very protrusile and can be subdivided into three subtypes: a) thick and muscular tongues as required for extraction of seeds from cones and husks such as in parrots, finches, and crossbills; b) tongues with extensive sharp papillae to hold slippery preys such as in fish-eating birds or raptors; or c) tongues rich in thread-like papillae for aquatic filter feeding such as in waterfowls. In ducks, for example, the wide and fleshy tongue leaves no space free in the sides of the mandible mucosa and no taste buds are present there. In contrast, additional rostral fields of taste buds are located in the mandible under the tongue tip and in the palate and in both posterior and anterior parts of the oral cavity and include pressure buds in the tip of the bill and on the roof of the oral cavity (Berkhoudt, 1977; Fowler, 1991). In Mallards, taste buds occur not only on the tongue but also in the mouth floor and the bill tip. This suggests the ability of Mallards in distinguishing taste cues just by holding food materials between the bill tips without bringing them further into the oral cavity (Zweers and Wouterlood, 1973; Martin, 2017).
3 Type III has been identified for birds with long beaks and tongues. Tongues in this group are adapted to food collection being long and protrusile and functioning as probes. This is the case for woodpeckers, hummingbirds, honeyeaters, and lorikeets with tongues collecting sap, insects, or nectar. Species of this group have some areas at the back of their tongue and oropharynx containing high number of taste buds (Bath, 1906). A detailed and systematic study on oral distribution of taste buds in this group is missing.

### 3.3 Taste sensory cell types and comparative taste bud structure

**FIGURE 2:**
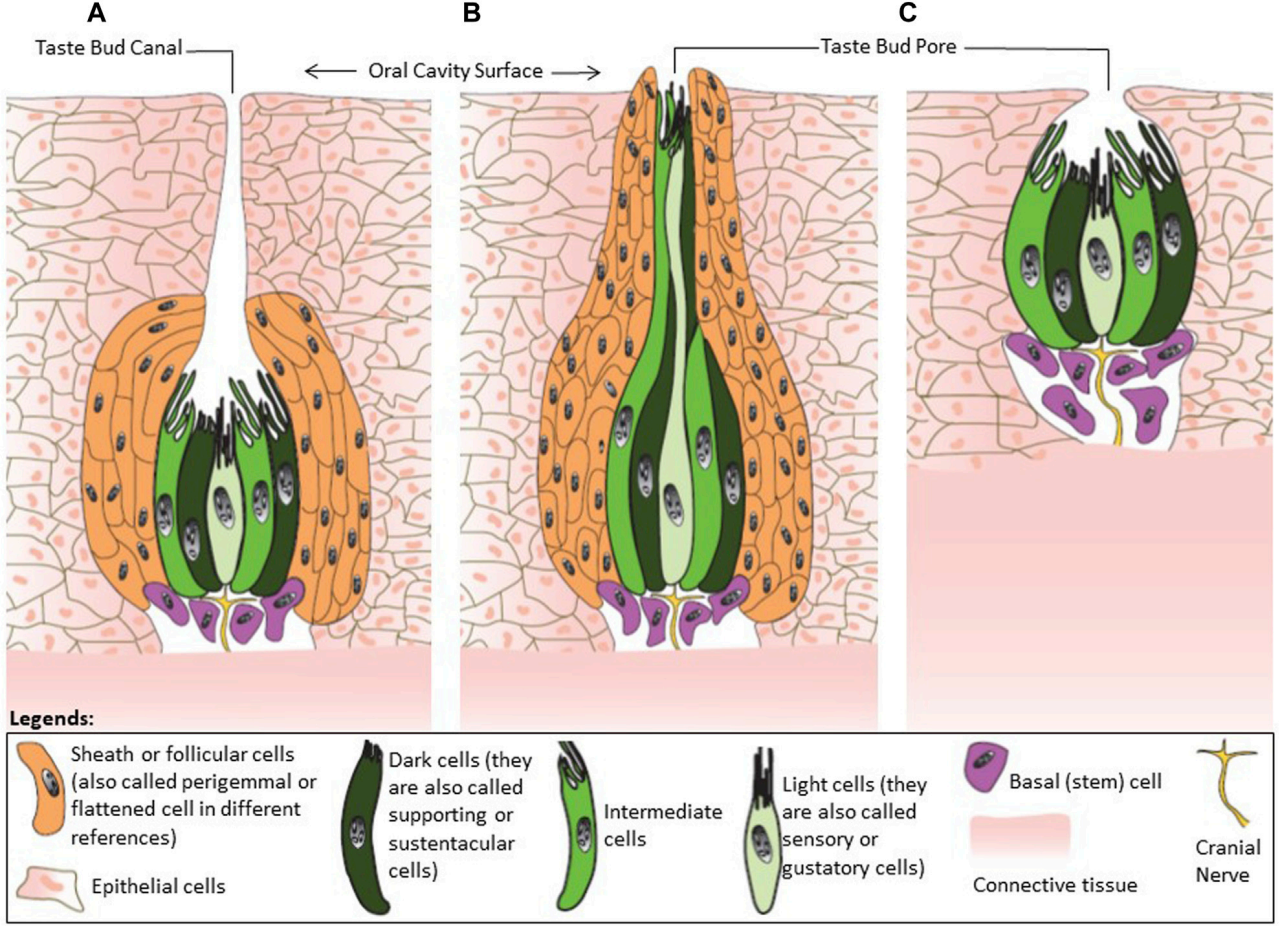
Classification of avian taste buds. **(A)** Type I is an ovoid taste bud enwrapped by follicular cells. **(B)** Type II have an elongated shape. **(C)** Type III has no follicular cells. This figure was created based on the descriptions from Bath (1906), Rowland et al. (2015) using Adobe Illustrator 24.0.

According to Berkhoudt (1992) avian taste buds consist of four types of cells including sensory cells (light cells in the electron microscope with a low-density cytoplasm and high number of vesicles), supporting cells (dark cells with a dense cytoplasm and less vesicles), follicular cells, and basal cells (Figure 2). Ganchrow and Ganchrow (1987) also found four different cells in chicken taste buds labelling as light, intermediate, dark, and basal cells. The light was identified as sensory cells, and dark and intermediate cells as supporting cells. In mammals, these different cells are commonly known as Type I, II, III, and IV (Chaudhari and Roper, 2010; Roper and Chaudhari, 2017). These four types are probably the equivalent of dark cell, light cell, intermediate cell, and basal cell, respectively (Figure 2). Type I and III in mammals would be the equivalent of the supporting cells in chicken’s buds (Kurosawa et al., 1983). Differences in cell types between avian and mammals is possibly related to the adaptation to nutrition requirements, feeding habits and pattern of ingesting food in birds.

In chickens, 55% of the cells in the taste buds belong to the Type I cell group referred to as dark cells (Ganchrow et al., 1998). These cells have a function similar to glial cells in the central nervous system where they clear the neurotransmitters from the extracellular environment, thus, terminating the signal transmission (Chaudhari and Roper, 2010). In addition, these cells are involved in the homeostasis of K^+^ in the buds and the transduction of salty taste (Vandenbeuch et al., 2008; Dvoryanchikov et al., 2009).

The light cells are equivalent to the Type II cells in mammals. They express the G-protein coupled receptors (GPCR) known to mediate sweet, umami, and bitter taste in humans (Roper and Chaudhari, 2017). Any single cell of this type expresses the receptors to convey only one taste type, i.e., either sweet, or bitter, or umami (Nelson et al., 2001).

The intermediate cells are equivalent to the mammalian Type III cells. They account for the lowest number of cells in the buds and are characterized by the synaptic junctions with neural fibers (also referred as pre-synaptic cells). These cells receive signals from Type II cells amplifying the response to a broad range of tastants (Tomchik et al., 2007; Chaudhari and Roper, 2010).

The basal cells are at the bottom of the taste buds, and they are undifferentiated cells. These cells are the precursors of the other three types of cells in the buds (Roper and Chaudhari, 2017).

The number of cells required to form a taste bud in avian species does not differ significantly from the numbers observed in mammals based on comparative size. The size of taste buds (height × width) in some granivore and insectivore such as pigeon, swallow, woodpecker, and greenfinch are about 75 µm × 44 µm. Omnivore birds such as chickens, sparrow, and starling have bigger taste buds (114 µm × 32 µm) (Bath, 1906). In humans and pigs, taste buds are 79 µm × 39 µm and 93 µm × 36 µm, respectively. Avian taste buds have been classified into three groups based on histological structure and are represented in Figure 2 (Bath, 1906; Botezat, 1906; Berkhoudt, 1992; Rowland et al., 2015; Cheled Shoval et al., 2022):

The type I are ovoid-shaped taste buds enwrapped by follicular cells (Figure 2. I). Chickens, pigeons, and songbirds have Type I taste buds. In chickens, there is a long canal (tubule) in the taste buds ending with a pore at the surface of the epithelium. This canal is a feature that has not been observed in any mammalian species known to date.

The type II are elongated and narrow taste buds with the follicular cells protruding into the epithelial surface (Figure 2. II). Examples of avian species presenting Type II taste buds are ducks and waders.

The type III are mammalian-like rounded taste buds lacking follicular cells (Figure 2. III). Parrots are an example of avian species with Type III taste buds.

The diversity in taste bud types across avian species has been associated with the adaptation to available foods and the food patterns summarized in Table 1 (Rowland et al., 2015). Bird species feeding mainly on dry foods such as insectivores such as European starlings, have developed small taste buds (123 × 38 μm). In contrast, aquatic birds (e.g., ducks) appear with the largest taste buds (130 × 60 μm). As shown in Table 1, omnivore birds such as emu (96 × 51 μm) have smaller taste buds than carnivore species such as European kestrel (148 × 45 μm). In general, the average size of taste buds in birds seem to be bigger than in mammals except for ruminant species. The size might be related to the number of cells shaping the taste bud.

**TABLE 1:** Food consumption patterns and the taste system of birds.

Diet type^a^	Taste bud number, type, and other features	Number of T2Rs	Behavioral response to different tastants	References
Insectivores	European starling	White-throated sparrow: 13	Great tit: Preference for sucrose. Large variability for chloroquine diphosphate but consumes prey secreting defensive bitter compounds	Toda et al. (2021), Espaillat and Mason (1990), Hämäläinen et al. (2020), Bath (1906)
	200, Type I, height × width of taste buds is 123 × 38 µm	Bar-tailed Trogon: 12		
	Blue tit	Rifleman: 9	European starling: Avoidance from tannic solution	
	24, Type I	European starling: 7		
Carnivores	European kestrel: unknown, Type I, height × width of taste buds is 148 × 45 µm	Owl: 2	Overall, this category of birds accepts sugary solutions	Abumandour and El-Bakary (2017), Espaillat and Mason (1990), Werner et al. (2008), Cheled-Shoval et al. (2017b), Bath (1906)
		Falcon: 2	Passerine: Preference for Alanine and MSG	
Granivores	Pigeon	Pigeon: 1	Canary: Preference for sucrose	Toda et al. (2021), Matson et al. (2004), Bath (1906)
	59, Type I, height × width of taste buds is 93 × 62 µm	Zebra finch: 8		
	Bullfinch	Medium ground finch: 11	Cockatiels: Avoidance from quinine	
	42, unknown			
Omnivores	Chicken	Chicken: 3	Overall, this category of birds accepts sugary solutions	Crole and Soley (2015), Cheled Shoval et al. (2022), Niknafs et al. (2022), Duncan (1962), Balog and Millar (1989), Brand et al. (2022), SpillariViola et al. (2008), Rajapaksha et al. (2016), Wang and Zhao (2015), Berkhoudt (1977), West et al. (2022)
	767, Type I, height × width of taste buds is 114 × 32 µm, and more than 66% of the taste buds on the palate and the rest on the base of the oral cavity	Duck: 4	Chicken: Preference for Alanine, Calcium, long chain fatty acid, and salt at 85-10 mM. Avoidance from quinine, acidic or alkalic solution at high concentration	
	Duck: Mallard	Turkey: 4	Ostrich: Preference for salt at 14/g/kg of feed	
	375, Type II, 130 × 60 µm unknown, Type II	Kea: 2	Blackbirds: Avoidance from tannic solution	
	Japanese Quail: Turkey	Crow: 10	Muscovy duck: taste cues affected tactile foraging behavior	
	62, unknown 200, unknown			
	Emu: unknown, Type I, height × width of taste buds is 96 × 51 µm			
Frugivores	Parrot		Overall, this category showed higher preference for hexose monosaccharide compared to sucrose	Rio et al. (1988), Martinez del Rio and Stevens (1989), Napier et al. (2013)
	350, Type III			
Piscivores		Dalmatian Pelican: 2		Davis et al. (2010), Wang and Zhao (2015)
		Great Crested Grebe: 2		
Molluscivores	Waders and Flamingos: unknown, Type II	American Flamingo: 2		Davis et al. (2010), Wang and Zhao (2015)
Nectarivores		Anna’s Hummingbird: 10	Hummingbirds, sugarbirds, sunbirds, honeyeater, white eye, and bulbul: Preference for sugar solutions	Toda et al. (2021), Jackson et al. (1998), Clark et al. (2015), Toda et al. (2021)

T2Rs, Bitter taste receptors; MSG, monosodium glutamate.

^a^ Klasing, 1998.

There are a few additional differences between mammalian and avian taste buds that include the life span and the embryonic tissues of origin. The average life span of chicken’s taste buds is 3–4 days whereas in laboratory rodents is around 10–12 days (Ganchrow et al., 1994). Thus, taste bud’s basal (stem) cells in birds undergo a more rapid development to meet the high turnover (Liu et al., 2018; Wang et al., 2019a). In addition, it has been reported that taste bud cells are not derived from neural crests but from mesenchymal cells with high migratory properties in chickens. In contrast, taste bud cells in mammals are from epithelial origin (Witt et al., 2000; Niknafs and Roura, 2018; Roura and Foster, 2018; Yu et al., 2021).

### 3.4 Neuroanatomy of the avian taste system

**FIGURE 3:**
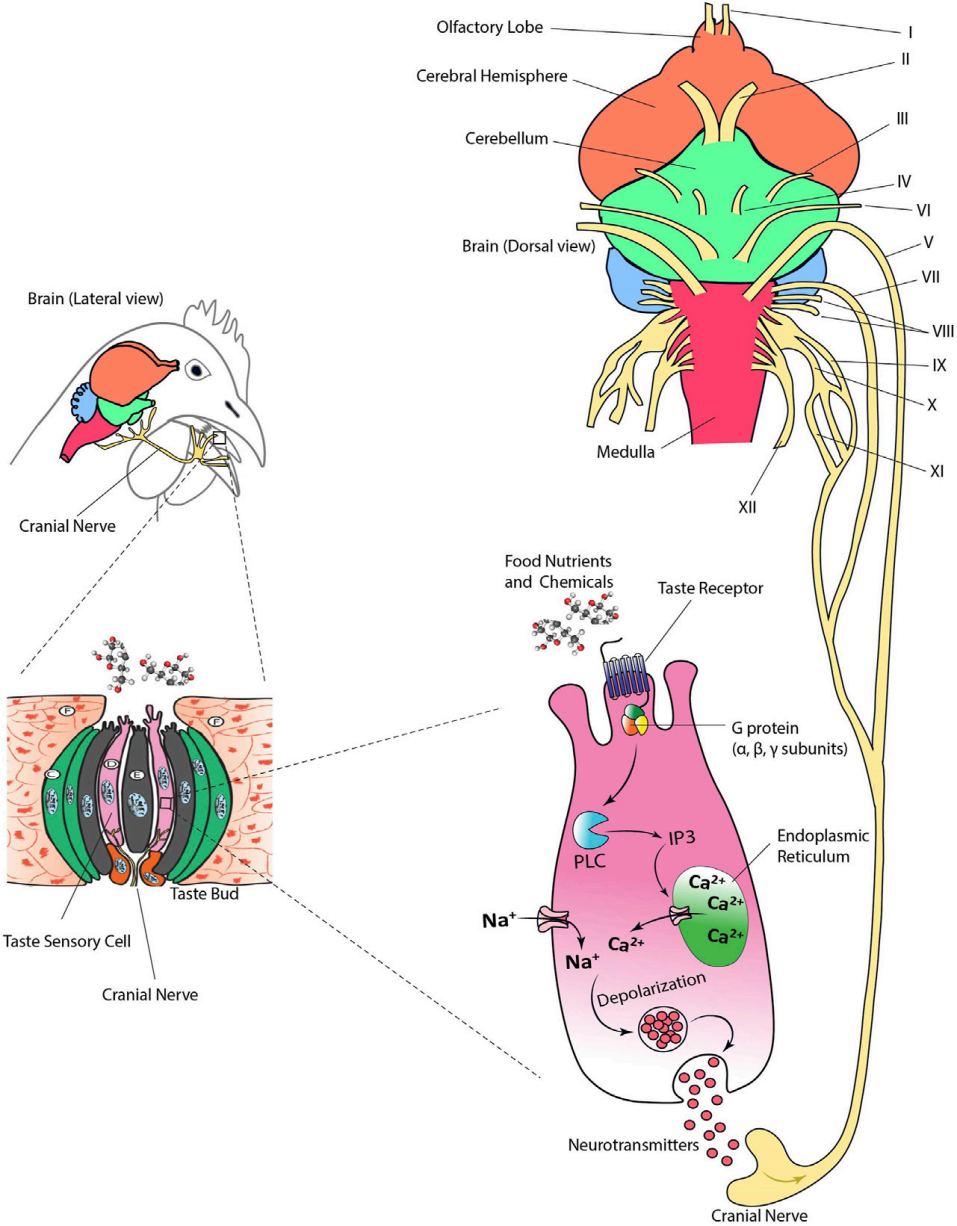
Taste transduction via cranial nerves in birds. Cranial nerves (CN) V, VII, IX, X, and XI transmit taste information from the taste buds to the brain. Sensory ganglia are not shown. Figure was created using Adobe Illustrator 24.0.

Nutrients and other chemical compounds present in food such as glucose, amino acids, minerals, and organic acids come into contact with taste sensory cells in the oral cavity. Dietary nutrients bind to the TRs and transmembrane channels triggering a cascade of biochemical reactions. These reactions trigger a signal resulting in the release of neurotransmitters and the excitation of cranial nerves that ultimately will stimulate the gustatory cortex of the brain (Figure 3). There are 12 cranial nerves (CN) in birds of which V, VII, IX, X, and XI are involved in transmitting taste information (Gentle, 1983; Clippinger et al., 1996; Orosz, 1996; Orosz and Bradshaw, 2007; Roper, 2007; Clark et al., 2015).

#### 3.4.1 CN V (Trigeminal Nerve)

This nerve has 3 main branches (V1, V2, and V3). The V3 branch is innervating sensory information from mucosa and skin at the lower bill and rictus (Orosz and Bradshaw, 2007). It also transmits gustatory (taste) information from the taste buds in the floor of the oropharynx to the nucleus solitaries of the medulla in the central nervous system (Orosz, 1996).

#### 3.4.2 CN VII (Facial Nerve)

It has been described that the facial nerve controls muscles responsible for facial expression in mammals. However, there is a poor development of facial expression with only one muscle, the mandibular depressor, innervated by CN VII in birds (Orosz and Bradshaw, 2007). In addition, CN VII is involved in transmission of taste information from the tongue to the nucleus solitaries of medulla in the central nervous system. The chorda tympani which is a branch of CN VII transmits information from the taste buds close to the anterior mandibular salivary glands. Most of the glands in the head including salivary glands, which are key elements in taste perception, receive parasympathetic intervention from CN VII (Berkhoudt, 1980; Gentle, 1984; Ganchrow et al., 1986).

#### 3.4.3 CN IX, X, XI (Glossopharyngeal Nerve)

These nerves are bundled together leaving the ventrolateral medulla. CN IX, X, and XI gather taste information from the posterior buccal and pharyngeal areas in avian species. CN IX and VII convey the signals from lingual taste buds and taste perception (Clippinger et al., 1996).

## 4 Taste sensing: physiology and feeding behavior

The taste receptors (TRs) involved in nutrient sensing are highly conserved in vertebrate animals and can be traced as far back as Dinosauria (Baldwin et al., 2014). The taste qualities that nutrients and other compounds may elicit in birds can only be inferred from the taste qualities known in humans (i.e., sweet, umami, bitter, fatty, salty, and sour). In birds, the family 1 taste receptors (T1R1 and T1R3) mediate the taste of amino acids and sugars, this is, umami and sweet taste in humans, respectively. The Family 2 referred to as the T2Rs, are associated with bitter perception. In addition, it seems that umami and bitter compounds stimulate different parts of the brain in chickens. Umami tastants triggered higher neural response (measured by c-Fos activity) in the right hemisphere of the nucleus taeniae of the amygdala, while bitter tastant created higher response in the left hemisphere (Protti-Sánchez et al., 2022).

The intracellular biomarkers specific to taste sensory cells originally identified in mammalian species (i.e., laboratory rodents or humans) are also highly conserved in birds. For example, the α subunit of the G-protein *α*-gustducin has been extensively used as a biomarker of taste sensory cells in chickens (Rajapaksha et al., 2016; Venkatesan et al., 2016) (Figure 3). Other cytosolic compounds identified as part of the taste transduction cascade in birds include PLC-β2 and TRPM5 (Witt et al., 1999; Venkatesan et al., 2016; Yoshida et al., 2018; Yoshida et al., 2019). In addition, vimentin has been identified as a taste sensory cell biomarker in chickens (Katsumoto et al., 1990). Vimentin is an intermediate filament protein involved in development of taste bud cells (Witt et al., 2000). This protein regulates cell migration and mechanics, and its expression is an indicator of transition from epithelial to mesenchymal cell (Sivagurunathan, et al., 2022). Thus, vimentin and *α*-gustducin seem to be specific to taste receptor cells in birds. However, recent data in chickens has shown that T1R3 is mainly expressed in vimentin-positive cells, while T2Rs were expressed in vimentin-negative cells indicating the specificity of taste-type marker of the filament (Yoshida et al., 2021a).

In recent years, significant progress has been made on how macronutrients such as carbohydrates, proteins, lipids, and calcium activate chemosensory mechanisms in the avian taste system (Niknafs and Roura, 2018). Each macronutrient has been related to one taste-type such as the umami to amino acids and peptides, sweetness to simple carbohydrates (sugars) and fatty to free fatty acids. However, the qualification of fatty taste in humans is still controversial (let alone in birds). Overall, birds appear to have an acute sense of taste allowing for the discrimination of dietary macronutrients (Roura et al., 2013).

### 4.1 Umami taste

Dietary protein and some amino acids are essential nutrients across most vertebrate species (Alagawany et al., 2020; Li and Wu, 2023). It appears that the ancestral T1R family of receptors evolved after the separation of vertebrates and invertebrates preceding the emergence of terrestrial animals. In particular, the heterodimer T1R1-T1R3 has become the principal dietary amino acid receptor in fish, amphibians, avian and mammalian species (Oike et al., 2007). In humans, the heterodimer was identified as the TR responsible for eliciting umami (savory) taste (Nelson et al., 2001). All avian species require dietary sources of amino acids such as meat, grains, or insects. Thus, it is likely that an umami-like taste has been developed in birds to identify food rich in amino acids (Rowland et al., 2015). The T1R1-T1R3 heterodimer has been found in all birds studied to date across all feeding styles including carnivores (e.g., falcon), piscivores (e.g., sea birds), micro-faunivores (e.g., some ducks), insectivores (e.g., swift, flycatcher), omnivores (e.g., quail), herbivores (e.g., some ducks), and granivores (e.g., chicken, finches, pigeons) (Shi and Zhang, 2006; Roura et al., 2008; Zhao et al., 2011). However, in addition to amino acids, the T1R1-T1R3 sensor in songbirds has mutated to sense simple sugars consistent with a feeding strategy specialized in sugar-rich food resources (Baldwin et al., 2014; Toda et al., 2021; Cockburn et al., 2022). The latter has been further elaborated in Section 4.2. The sensitivity of T1R1-T1R3 to amino acids and sugars changes across different bird species. Alanine, lysine, arginine, asparagine, valine, serine, and glycine trigger the T1R1-T1R3 heterodimer more than other amino acids (Cockburn et al., 2022).

Interestingly, L-alanine showed the highest affinity for the chicken T1R1-T1R3 consistent with the responses reported in other avian species like the swift, or other vertebrates such as the medaka fish or the mouse (Baldwin et al., 2014; Yoshida et al., 2022). Behavioral studies in passerines and chickens also showed robust taste preferences for alanine. Both red-winged blackbirds and starlings preferred Alanine solution at ≥0.7% concentrations (Espaillat and Mason, 1990; Werner et al., 2008; Niknafs et al., 2022). From a nutritional point of view, it is relevant to note that alanine is a non-essential amino acid (not required in feeds) since it can be synthesized sufficiently from metabolic precursors in all eukaryotic cells. Another non-essential amino acid eliciting robust preferences in birds is glutamic acid or monosodium glutamate (MSG). Glutamic acid is the most abundant amino acid in animal tissues, particularly muscle fibers (Dalle Zotte et al., 2020). The threshold of detecting MSG in chicken was reported at 300 mM indicating a lower sensitivity than humans with mean detection threshold of 1.22 mM (Cheled-Shoval et al., 2017b; Lim et al., 2022). However, some researchers have observed low preference for umami solution (Yoshida et al., 2021a). This has been associated with the findings that T1R1-T1R3 heterodimer is barely formed in chickens since T1R1 and T1R3 were only co-expressed in 5% of the taste sensory cells (Yoshida et al., 2021a). However, further data is needed to support such claim since the heterodimer was shown to be responsive to alanine and serine in a chicken cell model (Baldwin et al., 2014).

In addition to T1R1-T1R3, other amino acid or peptide receptors such as mGluR1, mGluR4, GPR92, CaSR, GPR139, and GPRC6A have also been reported to be functional in the avian oral cavity (Baldwin et al., 2014; Cheled-Shoval et al., 2014; Niknafs et al., 2018). For example, CaSR is expressed in chicken taste buds and its activity increased responding to alanine, tryptophane, and phenylalanine (Omori et al., 2022). However, low extracellular calcium negatively affected the activation of CaSR by these amino acids (Omori et al., 2022). Furthermore, CaSR may play an essential role in sensing calcium which is discussed in Section 4.4.

### 4.2 Sweet taste

Many different bird species rely on simple (i.e., glucose, fructose, sucrose) or complex (i.e., starch) carbohydrates as the main dietary energy sources including frugivore, granivore, and nectarivore species (Klasing, 1998). The main sensor for simple carbohydrates (sugars) was identified as the heterodimer T1R2-T1R3 in mammals which in humans has been described to mediate sweet taste perception (Zhao et al., 2003; Meyers and Brewer, 2008). However, it appears that the T1R2 gene has been lost in avian species (Shi and Zhang, 2006; Zhao et al., 2011; Lagerstrom et al., 2006). Birds descended from carnivorous theropod dinosaurs who had no essential needs for carbohydrates (Padian and Chiappe, 1998). Thus, as it has been also shown in mammalian carnivores, the loss of T1R2 in the avian ancestor would have been related to the adaptation of the taste system to a carnivore feeding regime (Nei et al., 2008). The loss of the sweet taste receptor T1R2 may have played a key role in the early evolution of avian species in adapting to ecological niches. However, subsequent evolution of some avian species drifted to feeding patterns involving sugar-rich sources (e.g., nectar or sweet fruits) as the principal nutrient sources which, in turn, resulted in the development of high preferences for sugars in hummingbirds, sugarbirds and sunbirds (Jackson et al., 1998; Clark et al., 2015; Toda et al., 2021). These findings suggest that alternative T1R2-independent mechanisms for sugar detection have evolved in these species. A series of mutations occurred in the umami taste receptor that shifted the sensitivity of the T1R1-T1R3 dimer to carbohydrate ligands in these avian species (Baldwin et al., 2014; Toda et al., 2021). Such shift conferred the ability to perceive sweet taste in almost half of the living bird species (i.e., songbirds) in the absence of T1R2 (Toda et al., 2021). Baldwin and co-workers (2014) discovered that hummingbirds regained sweet taste perception by mutating both T1R1-T1R3 subunits. The mutated receptor in hummingbirds strongly responded to sucrose, fructose, glucose, sorbitol, erythritol and the artificial sweetener sucralose while showing a loss of affinity to amino acids compared to chickens or swifts (Baldwin et al., 2014). The regaining of the ability to taste simple carbohydrates (sweet taste) has been shown to be stable despite frequent transitioning in some bird species from and to nectar feeding (Toda et al., 2021).

Interestingly, the concentration and ratios between sucrose, glucose and fructose in nectar varies greatly among flowers and seems to be relevant to determine species-specific preferences (Honda et al., 2010). Within nectarivore bird species, different preferences have been related to differences in the efficiency of intestinal hydrolysis and osmolality (Leseigneur and Nicolson, 2009; Medina-Tapia et al., 2012; Martínez del Rio et al., 2015; Koutsos et al., 2016). Frugivore birds exhibit lower appetites for sucrose compared to hexose monosaccharides, seemingly due to the lack of sucrase and/or the lower absorption rates (Rio et al., 1988; Martinez del Rio and Stevens, 1989; Napier et al., 2013). This variations in the type of sugar preferences have been associated with the pollinator role of birds which would influence nectar composition, concentration, and volume as an example of pollinator-plant co-evolution (Lotz and Schondube, 2006; Johnson and Nicolson, 2008). As in mammals (Sclafani et al., 1987), sex differences relevant to sugar appetites have been reported in nectarivores (Espaillat and Mason, 1990; Markman et al., 2006). Gut transit time of sucrose in Palestine sunbirds was longer in males (50 min) than females (30 min) (Markman et al., 2006). This may be linked to differences in taste sensitivity, energy requirements, and digestive capacity between male and female birds.

Insectivorous, omnivorous, and carnivorous birds reportedly accept sugary solutions. However, sensitivity and thresholds tests have not been performed in most avian taxa (reviewed by Clark et al., 2015; Rowland et al., 2015). The threshold of detecting sucrose in chicken was reported at 1 M indicating less sensitivity of chickens compared to humans which was reported to be 6.8–10.2 mM (Cheled-Shoval et al., 2017b; Petty et al., 2020). Studies testing glucose, fructose, sucrose, and artificial sweeteners such as saccharin in chickens have been contradictory (recently reviewed by Cheled-Shoval et al., 2017a; Rowland et al., 2015). Since the chicken T1R1-T1R3 dimer has high affinity for alanine and serine and no affinity for sugars, it is tempting to speculate that chickens may have a T1R-independent sweet perception such as in laboratory rodents where the sweet sensing of glucose or maltose originate in starch hydrolysis. The apical membrane of taste buds has been associated with disaccharidase activities and the transmembrane Sodium-Glucose Transporter 1 (SGLT1) (Sukumaran et al., 2016). Chickens show an intact mammalian-like disaccharidase-SGLT1 system which may also account for the T1R2-independent glucose and galactose sensing mechanism in the oral cavity (Higashida et al., 2022). Another possible mechanism could be related to the activity of SGLT1 in extra-oral tissues such as intestine. In mice, for example, it has been shown that preference for sucrose over artificial sweetener was mediated by duodenal neuropod cells (Buchanan et al., 2022). Neuropod cells differentiate between sugar and sweetener by eliciting different neurotransmission pathways using SGLT1 and sweet taste receptors (Buchanan et al., 2022).

### 4.3 Bitter taste

In birds as in mammals, the sensing associated with the activation of family two taste receptors (T2R) plays a primary defense function to prevent the ingestion of potential toxic compounds presumably by eliciting bitterness or a similar unpleasant sensation, showing behavior responses like head shaking, beak wiping and tongue and beak movements (Gentle, 1978). Birds learn to use the distastefulness associated with bitter compounds as a signal of toxicity (Skelhorn and Rowe, 2010). Such deterrence to bitter tastants (e.g., quinine, D-pulegone or garlic oil) has been used to protect crops and horticulture or to protect birds from toxic pesticide applications (Mastrota and Mench, 1995; Hile et al., 2004; Clapperton et al., 2012). Blackbirds and European starlings avoided the consumption of tannic solutions (0.5%–5%, which is in the range found in fruits and grains) over distilled water inferring they could perceive a bitter/unpleasant taste or smell (Espaillat and Mason, 1990). Starlings were more sensitive to tannic acid solution (0.5%–1%) compared to blackbirds (Espaillat and Mason, 1990). This may be reflecting the difference in their feeding habits meaning starlings consume less of foods containing tannin.

Bitter compounds relate to many different chemical categories including alkaloids (e.g., glycoalkaloids present in tubers, fruits, and seeds), isoprenoids (e.g., terpenes found in plants and insects), or phenylpropanoids (such as polyphenols present in fruits and cereals). Interestingly, some of these bitter compounds have beneficial biological functions such as antimicrobial, antioxidant, or digestive enhancing activities (Bernards, 2010). In fact, for non-toxic compounds there is a biphasic response to bitterness consisting of a first innate aversion followed by an adaptive behavior and acceptance as reported in insectivores, granivores and nectarivores (Fink and Brower, 1981; Marples and Roper, 1997; Johnson et al., 2006; Skelhorn, 2015). In nectarivores, bitter compounds may selectively encourage or discourage the consumption of specific plant nectars. For example, phenolic-rich dark nectar from *Aloe vryheidensis* attracted dark-capped bulbuls while repelling sunbirds (Johnson et al., 2006). In the case of great tits (*Parus major*), the large variability in their perception threshold for chloroquine diphosphate (0.01 to 8 mmol/L) did not impact their foraging choices regarding the consumption of preys secreting the bitter compound as a defensive mechanism. The energy status (body condition) and not the bitter compound seemed to be the main driver of prey consumption (Hämäläinen et al., 2020). The threshold of detecting quinine in chicken was reported at 0.3 mM indicating a similar sensitivity level to mammals (Cheled-Shoval et al., 2017b). However, the *in-vivo* threshold is often higher than the *in-vitro* threshold (Cheled-Shoval et al., 2017a).

Avian species have a wide range of the bitter taste receptor repertoires. The range goes from one T2R in the turkey to up to thirteen T2R in the white-throated sparrow (Table 1), indicating a dynamic role in evolution to adapt to new environments (Davis et al., 2010). Such variation may have had evolutionary implications for bird species. In episodes of expansion, large number of receptors allow the birds to detect and discriminate a wider range of bitter compounds, with higher feeding specialization in a particular ecological niche. In contrast, during contraction episodes of the T2R repertoire, there is a reduced ability to identify different bitter substances (Dong et al., 2009; Davis et al., 2010). It has also been speculated that the loss of T2Rs in birds, such as in chickens, is related to the overall reduction in genome size with no subsequent occurrence of gene expansion (Go, 2006). Wang et al. (2019b) stated that higher number of T2Rs in Anna’s hummingbird compared to its two close insectivorous relatives (chuck-will’s widow and chimney swift) suggested increased sensitivity to bitter nectars. Bitter and potentially toxic nectars probably played a key role in developing specificity of pollinators (Wang et al., 2019b). A positive correlation between the number of avian T2Rs and the abundance of toxins in their diets has been reported. Insects normally secret bitter compounds as a defense mechanism; thus, this could be a reason for higher number of T2Rs in insectivorous birds compared to others (Wang and Zhao, 2015).

The short T2R repertoire in chickens consists of three widely tuned T2Rs (T2R1, T2R2 and T2R7) capable of responding to most compounds known to be bitter to humans (Li and Zhang, 2013; Roura et al., 2013; Behrens et al., 2014; Cheled-Shoval et al., 2015; Hirose et al., 2015; Kawabata et al., 2019; Yoshida et al., 2019). Chicken T2Rs were mainly expressed in vimentin-negative taste cells suggesting there might be a different downstream molecule involved in transmitting bitter sensation compared to mammals (Kawabata and Tabata, 2022). Chickens were found to respond to quinine at a similar detection threshold than humans and rodents which is between 0.1 and 0.3 mM (Cheled-Shoval et al., 2017b). Similarly, cockatiels (*Nymphicus hollandicus*) have a quinine sensitivity comparable to humans and superior to other mammals (Matson et al., 2004). Behrens et al. (2014) concluded that a low number of functional T2R genes in birds is possibly compensated by a wider tuning specificity and a high ligand affinity (Behrens et al., 2014).

In chickens, T2Rs are expressed at least in three different oral tissues: the palate, the base of the oral cavity and the posterior tongue (Yoshida et al., 2019). However, oral taste-bud numbers differ between breeds. Broiler chickens (genetically selected for meat production) have a larger number of buds, and these buds are linked to a higher sensitivity to quinine compared to egg producing breeds (Kudo et al., 2010). Age is also associated with bitter sensitivity. Young chicks show more sensitivity to bitterness and higher expression of T2R compared to adult chickens (Ueda and Kainou, 2005; Dey et al., 2018). Concurrently, it appears that bitter taste sensitivity can be affected by genetic selection in broiler chickens. Modern broiler chickens compared to their ascendants showed higher sensitivity to bitterants (Yoshida et al., 2021b).

### 4.4 Salty taste

The intracellular and extracellular concentrations of electrolytes are critical for life and therefore tightly regulated. In birds, the main dietary cationic minerals are sodium and potassium, while the major anionic electrolyte is chloride. Salt (NaCl) is particularly critical with both deficiency as well as excess consumption being lethal. The presence of nasal salt gland in marine birds enable them to tolerate relatively high levels of NaCL by safely removing them from the body. In contrast, high NaCl solutions (i.e., 2% or more) are toxic to birds without salt glands (Mason and Clark, 2000). Salt sensing has been shown in many different types of birds, but thresholds may vary significantly across species. In non-marine birds the taste of salt triggers two divergent behavioral responses, depending on the concentration of the food and the sodium status of the animal. High concentrations are aversive, while low concentrations show high preference particularly in a sodium deficient status (Meyer et al., 1986). For example, chickens show preference for salt solutions between 85 and 100 mM, while reject solutions of 250 mM or higher (Duncan, 1962; Balog and Millar, 1989). Also, in a free-choice assay, ostrich chicks, provided with a range of flavored feed (salt, sweet, sour, bitter), significantly preferred salt-added feed (14 g/kg) over other flavors as well as the control (Brand et al., 2022). In this study, the control feed (mainly consist of barley, maize, and soybean meals) contained 4 g/kg of salt, and the highest level tested was 34 g/kg. Other studies on preference for salt solutions include blackbirds, European starlings, cockatiels, and pigeons and ranged from 0.1%–1% (Espaillat and Mason, 1990; Nakajima and Onimaru, 2006). It is possible that salt taste mechanism in birds is similar to mammals. Unlike other taste qualities that have a dedicated cells in taste buds, salt taste detection is mediated by multiple sensory cells. Also, salt taste requires movement of sodium into the taste cell facilitated by ENaC channel. The sodium influx depolarizes the cell resulting in neurotransmitter ATP release and electrical signal to the central nervous system (reviewed by Taruno and Gordon, 2023).

### 4.5 Sour taste

Acidity in foods relates to the concentration of H^+^ ions released during the ingestion process in the oral cavity. Sour foods are often associated to bacterial or yeast fermentation. Thus, the associated presence of potential pathogens in foods evokes a protective rejection response. However, the response to acidic foods depends on the avian species and age (Mason and Clark, 2000). Studies in chickens have shown that, overall, there is a tolerance for medium acidic or alkaline solutions but strong avoidance for extreme acid or alkaline solutions including organic acids such as citric acid (Fuerst and Kare, 1962; Gentle, 1972; Balog and Millar, 1989; SpillariViola et al., 2008). For example, in a two-choice test of control feed *versus* feed supplemented with 6% citric acids, birds consumed 35% less from the citric acid added feed (Balog and Millar, 1989). The main sour taste receptor is thought to be the dimer transmembrane proton channel Otopetrin-1 (OTOP1) that is highly selective for hydrogen ions in mammals (Teng et al., 2019; Zhang et al., 2019). The protein coding gene has also been mapped for many avian species and can be found in genomic databases such as NCBI. However, further functional evidence of this receptor in avian species is currently lacking. In a recent study, chickens’ OTOP1 showed a similar response to extracellular acids compared to humans (Tian et al., 2023). Interestingly, OTOP1 in chickens, humans, and four other vertebrates responded to alkaline pH 9.0, suggesting that this gene is also an alkali-activated channel (Tian et al., 2023).

### 4.6 Calcium taste

Calcium is the most challenging mineral in bird diets, being the most limiting nutrient in bird reproduction (Reynolds and Perrins, 2010). Firstly, birds may require calcium not only for bone formation but also for eggshell production. Secondly, it is a common behavioral trait in birds to provide calcium-rich feeds to their chicks immediately after hatching (Reynolds and Perrins, 2010). Thirdly, calcium requirement is extremely variable during the bird lifespan. Variations in dietary calcium requirements may increase up to 20-fold during oviposition in some avian species (Klasing, 1998). Lastly, many foods available to birds are likely to be deficient in calcium. For example, the amount of calcium in seeds (granivores) or insects (insectivores) is insufficient for egg laying forcing female birds to select calcium-rich foods as a supplement (Graveland and van Gijzen, 1994). It is important to note that calcium metabolism is tightly associated with phosphorus and vitamin D metabolism involving intestine, kidneys, and bones in avian species. Deficiency of circulating calcium increases parathyroid hormone resulting in bone resorption, renal excretion of phosphorus, and increased intestinal absorption of calcium (Li et al., 2016). Preference for calcium-rich foods such as snail or mollusc shells is particularly apparent in the evening allowing the acid conditions in the gizzard to dissolve the calcium source and spare the mobilization of bone during the overnight eggshell formation (Houston et al., 1995; Graveland, 1996). Thus, a physiological mechanism to taste dietary calcium seems particularly essential in birds.

On the one hand it seems speculative to define a sense of calcium taste in birds. On the other hand, birds show a high preference for diets containing high amounts of calcium (Reynolds and Perrins, 2010). Calcium-driven foraging by laying birds has been widely reported for several species including pheasants (Sadler, 1961), vultures (Mundy and Ledger, 1976), great tits (Graveland and Berends, 1997), and geese (Campbell and Leatherland, 1983). Leeson and Summers (1978) offered laying hens with diets containing two levels of calcium (131 vs. 4.7 g/kg) and protein and energy (107 g CP and 7.28 MJ/kg vs. 191 g CP and 12.82 MJ/kg) and a control diet (30 g/kg Ca, 171 g CP, 11.69 MJ/kg). They observed 7% less feed intake and better shell quality in birds receiving the former diets compared to the control in choice feeding experiment. It was suggested by the authors that reduced feed intake was linked to an specific appetite for calcium (Leeson and Summers, 1978). Similarly, broiler chickens were able to adjust the consumption of a calcium supplement to the calcium level in feed according to growth requirements (Wood-Gush and Kare, 1966; Joshua and Mueller, 1979; Wilkinson et al., 2014). For example, Wilkinson et al. (2014) provided broilers with two complete diets containing different levels of calcium (5 and 10 g/kg) and access to a separate source of calcium (CaCO3), and they found that broilers fed with diet containing 5 g/kg of calcium consumed significantly higher amount of CaCO3. The Calcium Sensing Receptor (CaSR), a GPCR related to some amino acid and calcium sensing in mammals, is expressed in chicken’s oral tissue (Kawabata et al., 2018; Omori et al., 2022). Also, using cell model and Ca^2+^ imaging, Omori et al. (2022) demonstrated that extracellular calcium and magnesium activate chicken CaSR. Thus, it seems plausible that the CaSR functions as a calcium sensing receptor in birds. However, further studies are needed to demonstrate if ligands of chicken CaSR can elicit behavioral responses in chickens or other avian species. Elucidating the mechanism of calcium taste in avian species will have important implications on feed intake regulation, reproductive and feeding behaviors, and egg and meat production.

### 4.7 Fatty acid taste

Similar to the calcium oral sensing, it is also speculative to define a sense of fatty taste in birds based on only indirect evidence such as choice feeding tests and the existence of the fatty acid (FA) receptors in the oral cavity. Behavioral studies regarding FA sensing in chickens have consistently shown preferences for long-chain FA-supplemented feeds (Furuse et al., 1996; Mabayo et al., 1996; Vermaut et al., 1997; Palomar et al., 2020). Different fat sources at inclusion rate of 6% with different amounts of free FA and various level of unsaturated to saturated FA ratios were added to laying hens’ diet. Results showed a higher preference for higher free FA and a stronger preference for saturated than unsaturated FA (Palomar et al., 2020). In addition, chickens did not show significant preferences for oleic acid (a mono-unsaturated FA) in choice feeding tests (Kawabata et al., 2021). In contrast, other FA including poly-unsaturated omega-3 eicosapentaenoic and docosahexaenoic acids, and omega-6 arachidonic acid, activated the FFAR4 in chickens (Kawabata et al., 2022). Furthermore, birds like mammals appear to differentiate the sensing of short from medium or long chain FA. Taken together, these findings suggest that FA sensing is likely as relevant in birds as it is in mammals.

Free fatty acid receptors (FFAR) 2, 3, 4, and FA transporter CD36 are expressed in the oral cavity of chickens (Colombo et al., 2012; Sawamura et al., 2015; Kawabata et al., 2019). Each receptor responds to different fatty acid chain lengths. FFAR2 and FFAR3 respond to short while FFAR4 to long chain FA (Roura et al., 2019). In contrast, it appears that the two-medium chain FFAR identified in mammals (FFAR1 and GPR84) are missing in the chicken genome (Meslin et al., 2015). This warrants further investigations particularly with the recent release of whole genome sequence of hundreds of avian species (Feng et al., 2020).

## 5 Conclusion and future directions

Birds have a well-developed gustatory system exquisitely adapted to ecological niches, nutritional needs, and available food sources. The ability to sense different tastes begins before hatching and the rapid developments occurring during the peri-hatching period. Generally, it seems that birds compared to mammals have more diversity in terms of anatomical structures of taste buds with at least three identified types. Also, the distribution patterns of taste buds in avian species compared to mammals are more diverse and mainly located on palates instead of tongue. The interchange between umami and sweet tastes have played a key role in evolutionary process of avian species. Genetic mutations in umami receptors (T1R1-T1R3) have granted many bird species including songbirds the ability to sense sweet taste despite the loss of the relevant receptor (i.e., T1R2). Such genetic change has resulted in allowing avian species to develop unique feeding strategies (such as in hummingbirds). Although birds compared to mammals generally have lower number of bitter taste receptors, their receptors can detect a wider range of compounds. This may reflect higher diversity of natural diets available to birds.

Deeper knowledge of the avian taste sense, anatomical structure and post-ingestion consequences will improve our understanding of the feeding behavior and nutrient requirements of domestic and wild birds. Some of the data reviewed has shown the importance of revisiting previous findings. For example, the latest study reported 767 taste buds in the chicken oral cavity. This represents a high sensing capacity similar (when not superior) to mammalian species and clearly debunking previous assumptions and scientific reports inferring no or lower taste sensitivities in birds. More importantly, the recent release of genome sequences of hundreds of bird species as a part of the Bird 10,000 genomes (B10k) sequencing project will have great implications for not only studying individual birds but facilitating functional and taxonomical comparison between species. The availability of genomic sequences of taste receptors in different bird species provides the opportunity to study molecular structures and functions of taste receptors. Also, the genetic changes can be identified and accurately linked to dietary habits and ecological adaptations in avian species. Overall, this review brings together strong evidence suggesting the importance of taste system in avian species and fundamental differences compared to mammals. Large diversity of species and wide range of dietary habits in birds could be strongly linked to their taste system.
